# Predictors of tuberculosis incidence and the effects of multiple deprivation indices on tuberculosis management in OR Tambo district over a 5-year period

**DOI:** 10.1371/journal.pone.0264811

**Published:** 2022-03-10

**Authors:** Ntandazo Dlatu, Benjamin Longo-Mbenza, Teke Apalata

**Affiliations:** 1 Department of Public Health, Faculty of Health Sciences, Walter Sisulu University, Mthatha, South Africa; 2 Research Champion, Walter Sisulu University, Mthatha, South Africa; 3 Department of Laboratory Medicine and Pathology, Faculty of Health Sciences and National Health Laboratory Services (NHLS), Walter Sisulu University, Mthatha, South Africa; The University of Georgia, UNITED STATES

## Abstract

**Background:**

This study investigated the associations between socio-economic deprivation and tuberculosis (TB) treatment outcomes, alongside well-known TB risk factors. The effects of healthcare expenditures and their growth on trends in TB incidence from 2009 to 2013 were also assessed.

**Methods:**

Secondary data analysis was performed on data obtained from various sources including governmental, non-governmental and research institutions. Indicators for TB treatment outcomes included TB death rate, TB rate among the household contacts of the Index TB cases, TB treatment failure, HIV associated TB death rate, TB defaulter rate, and new TB smear positive cases. Analysis of variance (ANOVA) and Turkey’s tests for post-hoc analysis were used to compare means of variables of interest considering a type I error rate of 0.05. Regression models and canonical discriminant analysis (CDA) were used to explore the associations between trends in TB incidence and independent TB predictors. During CDA, Fischer’s linear functions, Eigen values, and Mahalanobis distances were determined with values of Wilk’s Lambda closer to zero being the evidence for well discriminated patient groups. Data analysis was performed using SPSS® statistical software version 23.0 (Chicago, IL).

**Results:**

In total, 62 400 records of TB notification were analyzed for the period 2009–2013. The average TB incidence rate over a 5-year period was 298 cases per 100,000 inhabitants per year. The incidence of TB was reduced by 79.70% at the end of the evaluation as compared to the baseline data in 2009. Multiple linear regression analysis showed that the Expenditure per patient day equivalent (PDE) and PHC expenditure per capita were significantly and independently associated with the decline of TB incidence (adjusted R^2^ = 60%; ρ = 0.002) following the equation: *Y = (- 209× Expenditure per PDE) + (- 0*.*191 × PHC expenditure per capita)*. CDA showed that in the most socio-economically deprived communities (quintile 1), HIV associated TB death rates were significantly more likely to be higher as compared to the least socio-economically deprived group (quintile 5) [Eigen value (12.95), function coefficient (1.49) > (.77); Wilk’s Lambda = .019, p < .0001].

**Conclusions:**

Although TB control programs in OR Tambo district have averted thousands of TB incident cases, their effects on HIV associated TB deaths among the most deprived communities remain insignificant. There is an urgent need for strengthening integration of TB/HIV services in most deprived settings.

## Background

According to the 2020 Global Tuberculosis (TB) report, almost 10 million new cases of TB disease were reported worldwide, and this was equivalent to 130 cases per 100,000 populations in 2019 [1]. Ranking above HIV/AIDS as the leading cause of death due to a single infectious agent, TB is confirmed to be among the top 10 killers globally [1, 2]. South Africa remains among the 30 high TB burden countries accounting for about 86% of all new cases of TB disease in the world [1]. The TB incidence in South Africa is estimated to 615 cases per 100,000 populations, ranging from 427 to 835 cases per 100,000 populations in 2019 [1]. This is despite the fact that TB incidence in South Africa has been drastically decreasing since 2009 [3]. Among the key driving factors, we have high rate of HIV co-infection, poverty and the emergence of multidrug-resistant TB (MDR-TB) and extensively-drug resistant TB (XDR-TB) [1, 3].

Health expenditure, expressed as total percentage (%) of the gross domestic product (GDP) in South Africa was reported at 8.797% in 2014, according to the World Bank [4]. However, its impact on trends of TB incidences in the country is not well documented.

Studies have shown that presently, KwaZulu-Natal, Eastern Cape, and Western Cape have the highest TB incidence rates in South Africa [5, 6]. Eastern Cape remains one of the poorest South African provinces characterized by disconnected and inadequate healthcare services. In recent years, attention has turned to the contributions made by the mismanagement of healthcare expenditures and poor service delivery by local governments (according to unverified reports) and the index of multiple deprivation in fueling both TB and HIV epidemics [5, 6]. Unconfirmed evidence also suggests that in the OR Tambo district of the Eastern Cape SA province, the expansion of TB services for people living with HIV has been slower particularly among communities living under extreme poverty. Although the World Health Organization (WHO) and the South African National Department of Health (NDoH) have issued strategic guidelines for the integration of TB and HIV services, yet these guidelines remain poorly implemented, especially among the most socio-economically deprived areas [7, 8]. Hence, the present study sought to investigate the associations between socio-economic deprivation and TB treatment outcomes, alongside other TB risk factors such as population density, number of people living in poverty, poverty gap, supervision rate in primary healthcare (PHC) facilities, and PHC work load. In addition, the effects of healthcare expenditures (Expenditure per capita, Expenditure per patient day equivalent–PDE, and the local government expenditure) and their growth over the years on trends of TB incidences over a 5-year period were also assessed.

## Methods

### Description of the study settings

**Map 1:**
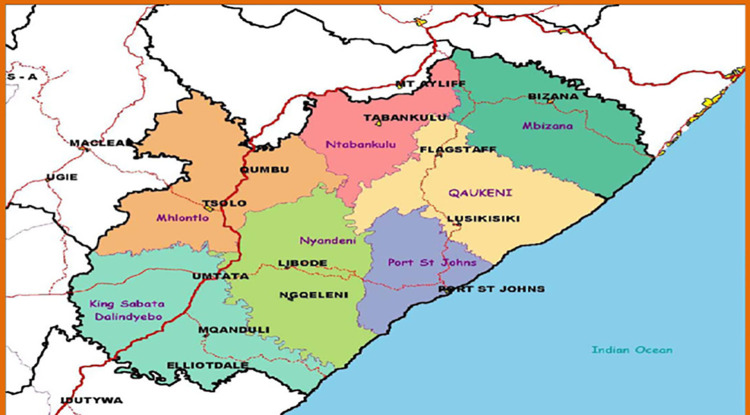
Map of OR Tambo district municipality. Source: http://isrdp.dplg.gov.za/documents/IDP/ISRDP/OR_Tambo_IDP.pdf. (open access).

O.R Tambo district (see Map 1) is one of the 7 districts of the Eastern Cape province of South Africa. The seat of O.R Tambo is in Mthatha. The vast majority of its population of about 1 676 463 speak isi-Xhosa [9].

The district is made up of 4 health sub-districts: King Sabata Dalindyebo sub-district with a population of 442318, Mhlontlo sub-district and its population is 221827, Nyandeni sub-district and its population is 436813, and Qaukeni sub-district with a total population of 659431 [9]. OR Tambo district has been reported to bear the following basic indicators: 64.6% of people are living under poverty with an estimated unemployment rate of 65.5% and the literacy rate of 42.2%. The average annual income of a Black resident is R15,762. Health services are delivered by one central hospital, 1 regional hospital, 12 district hospitals; 11 community health centers, 49 clinics, 52 health posts and 15 mobile health services [9].

### Study design, data sources and description of variables of interest

Secondary data review was performed on the OR Tambo TB data obtained between 2009 and 2013 from various sources including governmental, non-governmental and research institutions. Those included data from the 2009–2014 District Health Barometer by the Health System Trust (HST); the 2017 Global Monitoring Report by the WHO and the World Bank; the 2015 WHO End TB Strategy; the 2014–2015 Eastern Cape Department of Health Annual Reports and TB health records from healthcare facilities in the OR Tambo district; the 2015 Re-engineering of Primary Healthcare Strategy by the EC DoH; the 2017 Eastern Cape Socio-economic Consultative Council (ECSECC); the 2012 South African National HIV Prevalence, Incidence and Behaviour Survey by the Human Sciences Research Council (HSRC); the 2015 Guide to Monitoring and Evaluation for collaborative TB/HIV Activities by the WHO and the Global Funds; the 2015–2016 Adequacy and Efficiency in PHC Financing by the SA National Treasury and the National Treasury Report 2015/2016; and the 2017/2018 to 2019/2020 Annual Performance Plan (APP) by the SA NDoH.

**The following variables of interest were extracted from the above data sources**:

**Table:** 

**Variables**	**Definitions**	**Data sources from which the variables were extracted**
**a) Description of independent variables:**		
Index of multiple deprivation	It is defined as the measure of deprivation. This is essentially the measure of poverty. The deprivation index and socio economic quantiles comprise a new index of multiple deprivation developed by Noble et al. (2013), according to basket of variables from South African Census 2011 and South African Index of Multiple Deprivation (SAIMD) with four domains: income and material deprivation, employment deprivation, education deprivation, and living environmental deprivation, individual or household using equal weights in terms of socio economic quantiles (SEQ): SEQ 1st = most deprived, SEQ 2 = deprived, SEQ 3 = intermediate, SEQ 4 = well off, and SEQ 5 = least deprived [10].	• Statistics South Africa. 2017. Living Conditions of Households in South Africa, Pretoria: Statistics South Africa, p.13. Available at: http://www.statssa.gov.za/publications/P0310/P03102014.pdf. • World Health Organization/World Bank Group. 2017. Tracking universal health coverage: 2017 global monitoring report. Geneva: WHO; 2017 (http://apps.who.int/iris/bitstream/handle/10665/259817/9789241513555-eng.pdf, accessed 21 June 2018).
Population density	It is a measurement of the number of people in an area (Number of individuals per square kilometer). It is calculated by dividing the number of people by area.	• National Department of Health. 2017. National Indicator Data Set. Pretoria: NDoH; April 2017. • Statistics South Africa. 2019. Quarterly Employment Statistics Media Release. Available at http://www.statssa.gov.za/?p=12246 References.
People living in Poverty	The number of people living below US $1.90 a day according to the 2015 definition by the World Bank (this was equivalent to $1.25 a day in 2008). This new international poverty line defined in October 2015 by the World Bank represents a value below which there is absolute poverty. Poverty is state or condition in which people lacks financial resources, essentials for a minimum standard of living.	• Statistics South Africa. 2017. Labour Market Dynamics in South Africa, Report No. 02-11-02 • (2017), Pretoria: Stats SA, pp.6, 57. • The 2017 Global Monitoring Report by the WHO and the World Bank. • The 2015 Re-engineering of Primary Healthcare Strategy by the EC DoH. • The 2017 Eastern Cape Socio-economic Consultative Council (ECSECC).
Poverty gap index	The ‘poverty gap index’ takes the mean shortfall from the poverty line, and divides it by the value of the poverty line. It tells us the fraction of the poverty line that people are missing, on average, in order to escape poverty. It is simply given as the percentage of population that is below the poverty line.	• World Health Organization/World Bank Group. 2017. Tracking universal health coverage: 2017 global monitoring report. Geneva: WHO; 2017 (http://apps.who.int/iris/bitstream/handle/10665/259817/9789241513555-eng.pdf, accessed 21 June 2018). • Statistics South Africa. 2017. Labour Market Dynamics in South Africa, Report No. 02-11-02 • (2017), Pretoria: Stats SA, pp.6, 57. • The 2017 Global Monitoring Report by the WHO and the World Bank. • The 2015 Re-engineering of Primary Healthcare Strategy by the EC DoH. • The 2017 Eastern Cape Socio-economic Consultative Council (ECSECC).
Supervision visit rate in primary healthcare (PHC) facilities	It is obtained by dividing the actual number of official visits performed to a PHC by a clinic supervisor to the intended number of official visits set at the beginning of the year or financial year. It aims to promote continuing improvement on the performance of health workers’ by ensuring that objectives of health programs are adequate by managing difficulties encountered by staff, motivating staff and by improving staff performance, including continuing education and planning for training.	• District Health Barometer 2013/14. Durban: Health Systems Trust; October 2014. • 2009–2013 District Health Barometer by the Health System Trust (HST).
PHC work load	An estimate of the patient care-related nursing workload, determined by dividing the total sum of the nursing intensity points of patients by nursing resource units of the day. Its purpose is to analyse utilisation patterns, efficiency and equity in terms of staff distribution.	• District Health Barometer 2013/14. Durban: Health Systems Trust; October 2014. • 2009–2013 District Health Barometer by the Health System Trust (HST). • World Health Organization and the United Nations Children’s Fund (UNICEF). 2018. Available: https://www.who.int/docs/default-source/primary-health/declaration/gcphc-declaration.pdf.
PHC Expenditure per capita	It is the total amount spent per person uninsured by medical aid at PHC excluding district health management and District hospitals. It is important measure of equity in resource distribution. It is measured by dividing the total amount of money spent annually by each district by the total population in the district [11].	• District Health Barometer 2013/14. Durban: Health Systems Trust; October 2014. • The 2009–2013 District Health Barometer by the Health System Trust (HST). • The 2015–2016 Adequacy and Efficiency in PHC Financing by the SA National Treasury and the National Treasury Report 2015/2016. • The 2017/2018 to 2019/2020 Annual Performance Plan (APP) by the SA NDoH.
Expenditure per patient day equivalent (PDE)	Expenditure per patient day equivalent is a composite process indicator that connects financial data with service-related data from the hospital admissions and outpatient records. This indicator measures how the resources available to the hospital are being spent, and is a marker of efficiency.	• District Health Barometer 2013/14. Durban: Health Systems Trust; October 2014. • The 2009–2013 District Health Barometer by the Health System Trust (HST). • The 2015–2016 Adequacy and Efficiency in PHC Financing by the SA National Treasury and the National Treasury Report 2015/2016. • The 2017/2018 to 2019/2020 Annual Performance Plan (APP) by the SA NDoH.
Local government expenditure	A total amount of money spent on district health services per person without medical scheme coverage.	• District Health Barometer 2013/14. Durban: Health Systems Trust; October 2014. • The 2009–2013 District Health Barometer by the Health System Trust (HST). • The 2015–2016 Adequacy and Efficiency in PHC Financing by the SA National Treasury and the National Treasury Report 2015/2016. • The 2017/2018 to 2019/2020 Annual Performance Plan (APP) by the SA NDoH.
**b) Description of dependent or outcome variables:**		
TB death rate	It is defined as the estimated number of deaths due to TB, in one year per 100,000 populations. In this study, the obtained TB death rate was converted in proportion (%).	• The 2015 WHO End TB Strategy. • The 2014–2015 Eastern Cape Department of Health Annual Reports and TB health records from healthcare facilities in the OR Tambo district. • The 2015 Re-engineering of Primary Healthcare Strategy by the EC DoH. • District Health Barometer 2013/14. Durban: Health Systems Trust; October 2014. • 2009–2013 District Health Barometer by the Health System Trust (HST).
Index TB case	It is defined as the first culture-confirmed TB patient who already had at least two acid-fast bacilli (AFB)-positive smears [12]. All smear-positive pulmonary TB cases are considered as index TB cases and their contacts should be evaluated for TB. Hence, Index TB case can also be expressed as proportion (%).	• The 2015 WHO End TB Strategy. • The 2014–2015 Eastern Cape Department of Health Annual Reports and TB health records from healthcare facilities in the OR Tambo district. • The 2015 Re-engineering of Primary Healthcare Strategy by the EC DoH. • District Health Barometer 2013/14. Durban: Health Systems Trust; October 2014. • 2009–2013 District Health Barometer by the Health System Trust (HST).
TB treatment failure	This is a TB patient whose sputum smear or culture remained positive at 5 months or later following anti-TB treatment [12].	• District Health Barometer 2013/14. Durban: Health Systems Trust; October 2014. • 2009–2013 District Health Barometer by the Health System Trust (HST). • TBfacts.org. TB Statistics- incidence, prevalence, high burden, TBfacts.org, [online]. Available at www.tbfacts.org [Accessed 24 JANUARY 2021].
HIV associated TB deaths	Cases or proportion (%) of HIV-positive patients co-infected with TB (either bacteriologically confirmed or clinically diagnosed) and who died from HIV associated TB [13].	• The 2012 South African National HIV Prevalence, Incidence and Behaviour Survey by the Human Sciences Research Council (HSRC). • The 2015 Guide to Monitoring and Evaluation for collaborative TB/HIV Activities by the WHO and the Global Funds.
TB incidence	This is an estimated number of new cases of TB disease per 100,000 populations over one-year period.	• The 2015 WHO End TB Strategy. • The 2014–2015 Eastern Cape Department of Health Annual Reports and TB health records from healthcare facilities in the OR Tambo district. • 2009–2013 District Health Barometer by the Health System Trust (HST).
TB defaulter rate	It is the proportion of TB patients whose treatment was interrupted for 2 consecutive months without medical approval and their sputum remains positive [14]. Defaulter rate is measured by dividing the number of patients who defaulted TB treatment in a given year to the total number of cured patients from TB in the same year.	• The 2015 WHO End TB Strategy. • The 2014–2015 Eastern Cape Department of Health Annual Reports and TB health records from healthcare facilities in the OR Tambo district. • The 2015 Re-engineering of Primary Healthcare Strategy by the EC DoH. • District Health Barometer 2013/14. Durban: Health Systems Trust; October 2014. • 2009–2013 District Health Barometer by the Health System Trust (HST).
New TB smear positive cases	TB patients who have never had treatment for TB or who have taken TB drugs for less than 4 weeks whose sputum microscopy test (acid fast bacilli) is positive in at least one sputum sample [14].	• The 2015 WHO End TB Strategy. • The 2014–2015 Eastern Cape Department of Health Annual Reports and TB health records from healthcare facilities in the OR Tambo district. • The 2015 Re-engineering of Primary Healthcare Strategy by the EC DoH. • District Health Barometer 2013/14. Durban: Health Systems Trust; October 2014. • 2009–2013 District Health Barometer by the Health System Trust (HST).

### Data analysis and statistical methods

Continuous indicators of TB management were presented as mean ± standard deviation (SD) for bivariate analysis. Pearson’s correlation coefficient was used to measure the degree of linear association between variables in general and between TB indicators and deprivation-concentration in particular, ranging in magnitude on interval scale from -1 to +1. Analysis of variance (ANOVA) and Turkey’s post-hoc tests were used to compare means of variables of interest considering a type I error rate of 0.05. Regression models were used to explore the associations between trends in TB incidence and independent TB predictors. The regression coefficient represented the increase or decrease in the absolute magnitude of the independent variable for each unit of increase in the dependent variables using the equation: *Y = constant (slope) ± ax*. The slope index of inequality represented the linear regression coefficient that showed the relationship between the level of implementation, the burden of TB and deprivation. Canonical discriminant analysis (CDA) was used to identify the variable of interest that significantly discriminated different levels of socio-economic deprivation from 2009 to 2013. The major underlying assumptions of CDA were: (i) each predictor variable is normally distributed using histograms and P-P plots; (ii) there must be homogeneity of covariance across the groups–the Box’s M test of equality of covariance matrices was used to check the assumption of homogeneity of covariance across the groups using p < .001 as a criterion; (iii) there must be at least two groups or categories, with each study case belonging to only one group so that the groups are mutually exclusive and collectively exhaustive; (iv) the groups or categories should be defined before collecting the data; (v) the predictor variable(s) used to separate the groups should discriminate quite clearly between the groups so that group or category overlap is clearly non-existent or minimal; (vi) group sizes of the dependents should not be grossly different and should be at least five times the number of independent variables.

In order to check for the assumptions (iii), (iv), (v) and (vi), we used Mahalanobis distances in order to support the classification of canonical variates into distinct groups and for comparing divergence among populations’ group centroids so that we can determine the degree of segregation with values of Wilk’s Lambda closer to zero being the evidence for well-separated groups. Each variable that is more than 1.96 Mahalanobis distance units away from a specific centroid group has less than 5% chance of belonging to that group. In addition, during CDA, Fischer’s linear functions and Eigen values were determined considering values of Wilk’s Lambda. Data analysis was performed using SPSS® statistical software version 23.0 (SPSS Inc; Chicago, IL).

### Ethics approval and consent to participate

The Research Ethics and Biosafety Committee of the Walter Sisulu University approved the study (ethical clearance No. 29/2014) and permission to conduct the study was obtained from the Eastern Cape Department of Health. This was a secondary data analysis, hence no consents from patients were obtained.

## Results

In total, 62 400 records for TB notification were analyzed for the period 2009–2013. The following findings were obtained:

### 1. Predictors of TB incidence in O.R. Tambo district

**Fig 1:**
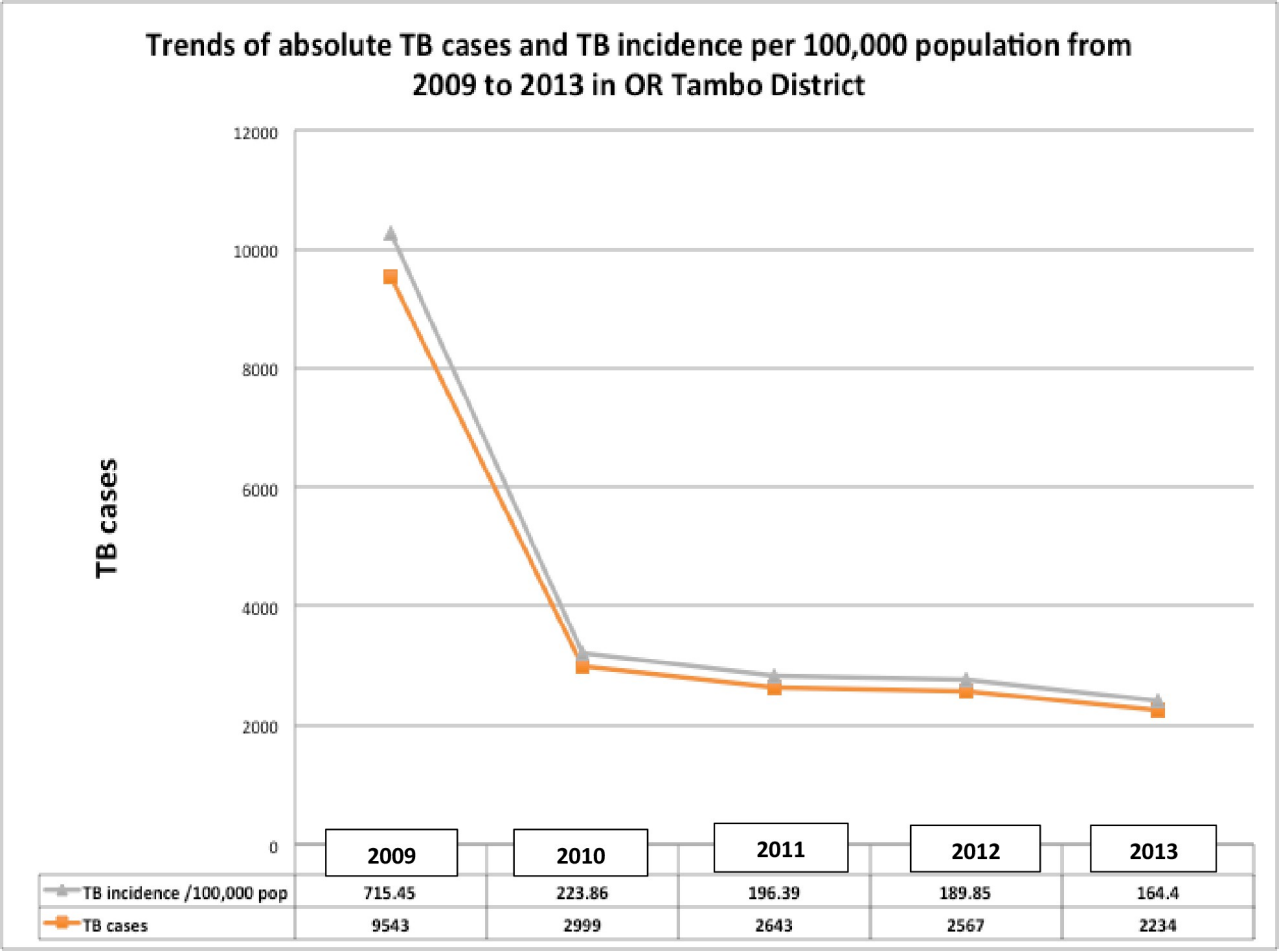
Trends of absolute TB cases and TB incidence per 100,000 population from 2009 to 2013 in OR Tambo district, Eastern Cape province–South Africa.

**Fig 2:**
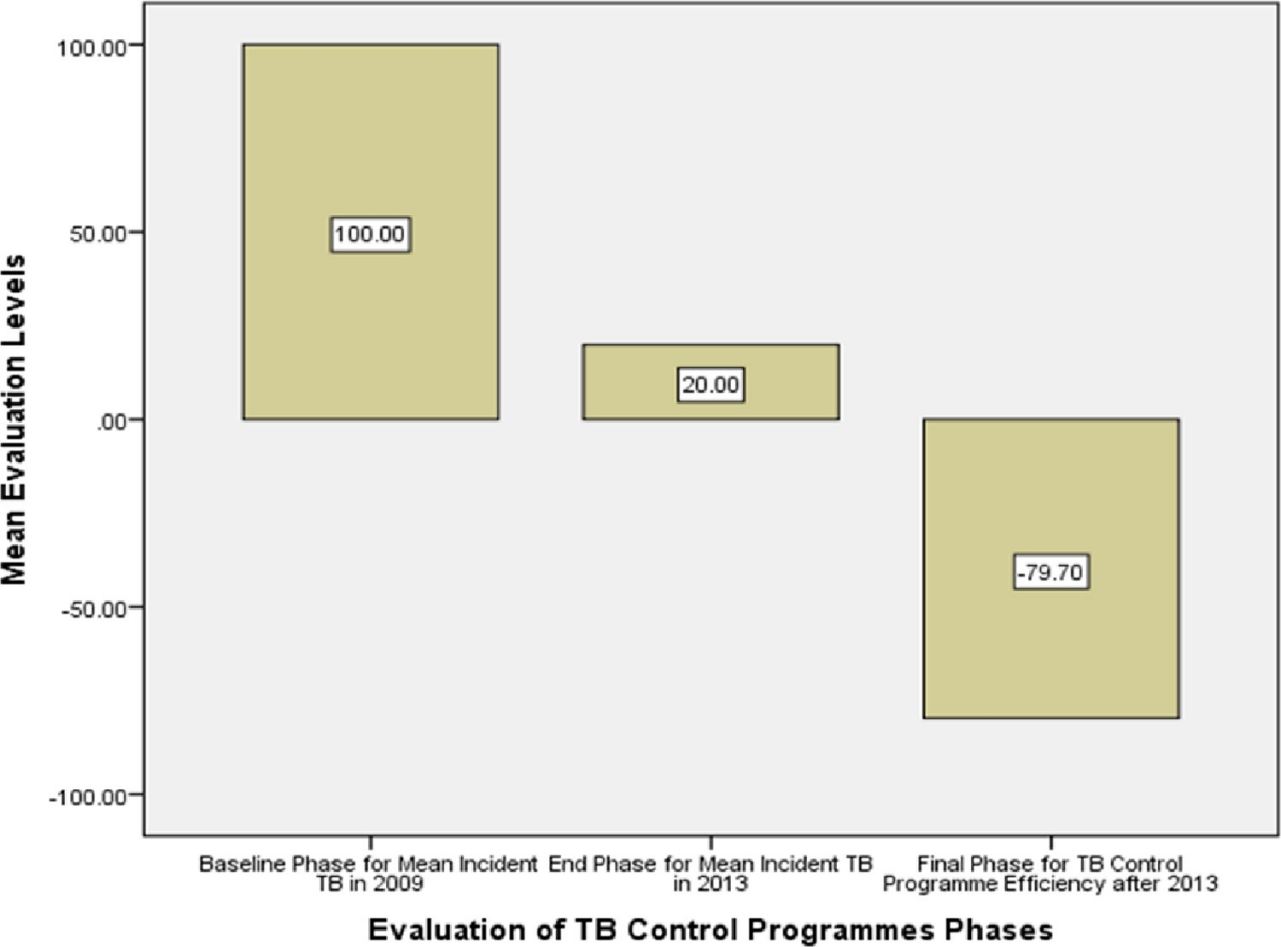
Performance efficiency evaluation of the implementation of TB control program displaying 79.7% reduction of TB incidence over a 5-year period.

**Table 1** and **Fig 1** display trends of TB incidence per 100,000 populations over a 5-year period as well as the actual TB cases diagnosed annually from 2009 to 2013. The incidence curve of TB trends dropped abruptly and significantly from 2009 until 2013 (ρ < 0.00001) (**Table 1** and **Fig 1**) as a result of a performance efficiency demonstrated by the reduction of 79.70% of TB incidence at the end of 2013 as shown in **Fig 2**.

**Table 1:** TB incidence and actual TB cases over a 5-year period (2009–2013).

Year	TB cases	Population	TB incidence per 100,000 inhabitants
2009	9,543	1,333,846	715.45
2010	2,999	1,339,680	223.86
2011	2,643	1,345,798	196.39
2012	2,567	1,352,097	189.85
2013	2,234	1,358,916	164.40

Data on population density and related poverty indicators as well as indicators for healthcare expenditures and their growth over a 5-year period are displayed in **Tables 2** and **3**, respectively. Correlation analyses between TB incidence rates and potential TB predicting factors in **Tables 2** and **3** showed a bivariate positive correlation between population density (r = 0.812; ρ < 0.0001), poverty gap index (r = 0.210; ρ < 0.01), and TB incidence (new cases of TB per 100,000 populations). However, there was a significant negative correlation between supervision rate (r = - 0.173; ρ = 0.030), PHC professional nurse clinical workload (r = -0.164; ρ = 0.021), Expenditure per patient day equivalent (r = -0.282; ρ = 0.015), PHC Expenditure per capita (r = -0.159; ρ = 0.034), local government expenditure on PHC (r = -0.244; ρ = 0.022), and TB incidence.

**Table 2:** Number of population density, people in poverty, poverty gap, and TB incidences between 2009 and 2013.

Year	Population Density	Number of people in poverty	Poverty gap (%)	TB incidence (per 100,000 populations)
2009	112.51	1,196,446	31.3	715.45
2010	113.61	1,183,517	32.8	223.86
2011	114.76	1,188,933	33.5	196.39
2012	115.84	1,186,284	35.2	189.85
2013	117.10	1,183,635	36.1	164.40

**Table 3:** Number of supervision rate, PHC workload, PHC Expenditure per PDE, PHC expenditure per capita and local government expenditure between 2009 and 2013.

Year	Supervision rate by district	PHC workload	PHC expenditure per capita (Rands)	Expenditure per PDE (Rands)	Local government expenditure (Rands)
2009	50.0%	19.5%	500	543	569.3
2010	87.3%	16.9%	500	1400	685.4
2011	89.6%	39.8%	595	1597	673.6
2012	83.0%	43.7%	626	1645	666.8
2013	80.8%	45.7%	647	1645	646.6

However, using a multiple linear regression analysis, only Expenditure per patient day equivalent (PDE) and PHC Expenditure per capita were identified as the most important, significant and independent predictors (adjusted R^2^ = 60%; ρ = 0.002) in terms of declining TB incidence following the equation: *Y = (- 209× Expenditure per PDE) + (- 0*.*191 × PHC expenditure per capita)*.

### 2. Association between socio-economic deprivation and TB care indicators in O.R. Tambo district

**Fig 3:**
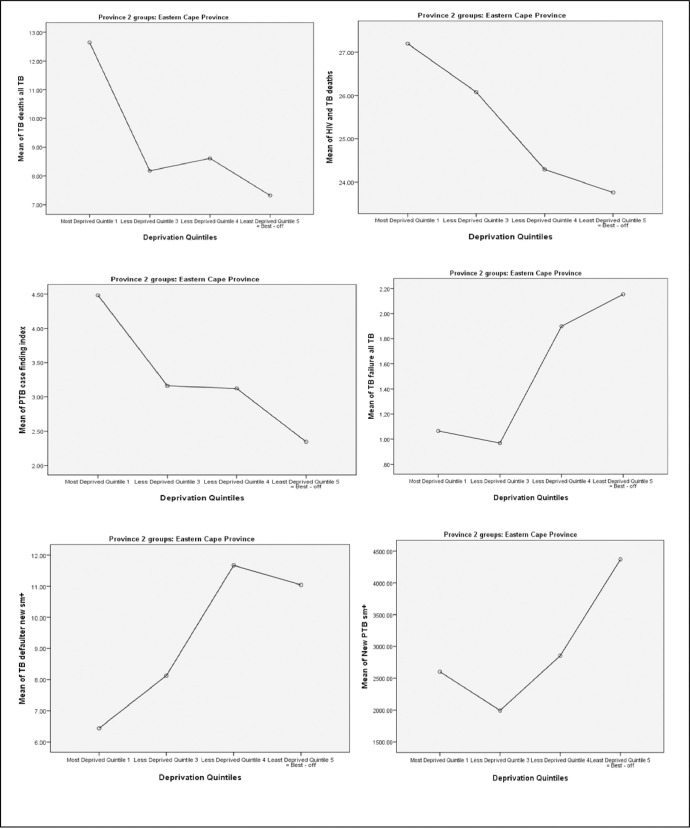
Associations between socio-economically deprived groups and TB care indicators in OR Tambo district over a 5-year period (2009–2013). A. Correlation between TB death rate and deprivation index (P < 0.0001). B. Correlation between HIV associated TB death rate and deprivation index (P = 0.049). C. Correlation between TB rate among household contacts of index TB cases and deprivation index (P < 0.0001). E. Correlation between TB treatment failure rate and deprivation index (P < 0.0001). D. Correlation between TB defaulter rates and deprivation index (P < 0.0001). F. Correlation between new TB smear positive cases and deprivation index (P = 0.001).

**Table 4** shows bivariate associations between TB care indicators and levels of socio-economic deprivation over a 5-year period. Following Turkey’s tests for post-hoc analysis, significantly higher TB death rates were observed in the most socio-economically deprived group (Quintile 1) as compared to Quintile 3, Quintile 4, and the least socio-economically deprived group (Quintile 5) (P < 0.0001) as shown in **Fig 3A**. Although a higher HIV-associated TB death rate was also observed among the most socio-economically deprived population (Quintile 1) than other socio-economically deprived groups, this difference was significant only when comparing Quintile 1 and Quintile 5 (P = 0.02) (**Fig 3B**) during a post-hoc multiple comparisons. TB incidence rates among the household contacts of the index TB cases were significantly more likely to be higher in the most socio-economically deprived group (Quintile 1) as compared to Quintile 3 (P = 0.007), Quintile 4 (P = 0.005), and the least socio-economically deprived group (Quintile 5) (P < 0.0001) (**Fig 3C**).

**Table 4:** A 5-year average (mean ± SD) notification of TB indicators by levels of socio-economic deprivation from 2009 to 2013.

TB control indicators	Quintile 1 (most deprived)	Quintile 3	Quintile 4	Quintile 5 (least deprived)	ANOVA P-value
TB death rate	12.64 ± 0.99	8.18 ± 0.25	8.61 ± 0.74	7.32 ± 0.45	<0.0001
HIV-associated TB death rate	27.19 ± 5.39	26.08 ± 5.31	24.30 ± 4.09	13.76 ± 4.37	0.049
New TB smear positive cases	2602 ± 1074	1995 ± 406	2855 ± 425	4367 ± 144	0.001
TB rate among the household contacts of the Index TB cases	4.48 ± 0.92	3.16 ± 0.37	3.12 ± 0.29	2.39 ± 0.10	<0.0001
TB defaulter rate	6.98 ± 1.24	8.85 ± 0.33	11.53 ± 1.44	1.25 ± 0.56	<0.0001
TB treatment failure rate	1.06 ± 0.45	0.97 ± 0.10	1.90 ± 0.17	2.15 ± 0.28	<0.0001
TB and HIV co-infection rate	79.45 ± 11.8	75.40 ± 16.83	76.22 ± 14.9	71.05 ± 11.73	0.57
TB Rifampicin resistance rate	5.88 ± 1.91	0.89 ± 0.39	1.22 ± 2.53	0.35 ± 5.18	0.71

However, significantly higher TB defaulter rates were observed among individuals in Quintile 3 (P = 0.015), Quintile 4 (P<0.0001) and the least socio-economically deprived group (Quintile 5) (P<0.0001) as compared with the most socio-economically deprived group (Quintile 1) (**Fig 3D**). Similarly, higher TB treatment failure rates were significantly observed among the least deprived groups, particularly Quintile 4 and Quintile 5 groups (P<0.0001) as compared with the most socio-economically deprived group (Quintile 1) (**Fig 3E**). Finally, there was a significantly high number of new TB smear positive cases observed in the least socio-economically deprived group (Quintile 5) as compared to Quintile 3 (P = 0.001) and Quintile 1 groups (P = 0.002) as shown in **Fig 3F**.

Using canonical discriminant analyses (CDA) on normally distributed variables that showed significant univariate associations, three canonical functions were identified as able to discriminate among the socio-economically deprived groups. The obtained 3 discriminant functions had Eigen values that are summarized in **Table 5**. The larger the Eigen value represents more shared variance in the linear combination of variables.

**Table 5:** Eigenvalue functions, % of variance, Cumulative variance (%), and Canonical Correlations during a CDA.

Function	Eigen value	% of variance	Cumulative %	Canonical correlation
1	12.954	84.5	84.5	.964
2	2.142	14.0	98.5	.826
3	.232	1.5	100.0	.434

CDA: Canonical Discrimination Analysis.

Of the 3 identified canonical functions, the first two functions contributed a total of 98.5% of the total variance, which easily stratifies into the 5 study groups (from quintile 1 to quintile 5) (Box’s M (3.77), p (0.47) > α (0.001)). The Wilks’ Lambda value was significant: 0.019; χ^2^ = 137.62; ρ < 0.0001 (**Table 6**).

**Table 6:** Wilks’ Lambda with test of functions during a canonical discrimination analysis.

Test of Function(s)	Wilks’ Lambda	Chi-square	Df	Sig.
1 through 3	.019	137.615	15	.000
2 through 3	.258	46.681	8	.000
3	.812	7.188	3	.066

Functions’ coefficients were calculated and used to decide which variables predicted group membership. Comparing the values between groups, the higher coefficient means the predictor variable attributes more for that group (**Table 7**).

**Table 7:** Structure matrix following a canonical discrimination analysis.

TB control indicators	Functions		
	1	2	3
TB death rate	.763*	-.059	.161
TB rate among the household contacts of the Index TB cases	.302*	-.130	.246
TB treatment failure rate	-.230	.578*	.093
HIV-associated TB deaths	-.046	-.224*	-.169
TB defaulter rate	-.403	.378	.785*
New TB smear positive cases	-.087	.412	-.670*
TB and HIV co-infection rate	.123	.137	.211*

*Largest absolute correlation between each variable and any discriminant function.

**Fig 4:**
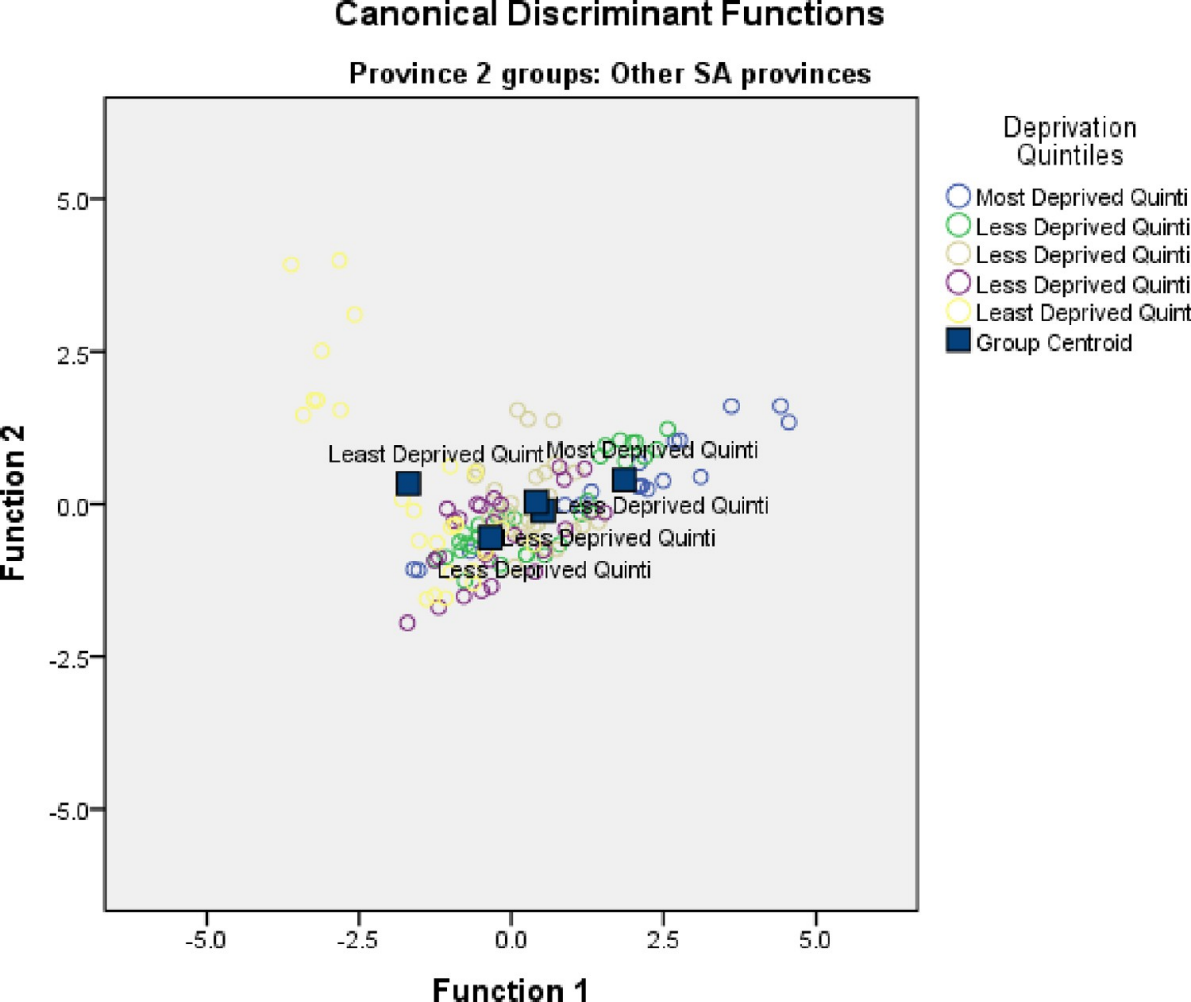
Canonical discriminant functions displaying socio-economic deprivation quintiles (Group centroids) discriminated by TB care outcomes between 2009–2013 in OR Tambo district.

**Table 8** demonstrated Fisher’s linear discriminant functions according to classification function coefficients. After adjusting for confounding factors (TB rate among the household contacts of the Index TB cases, TB defaulter rate, new smear positive TB cases, TB death rate, and TB treatment failure), only HIV-associated TB death rate was identified as the most important, significant, and independent indicator able to discriminate the most deprived communities with a deprivation index far from other deprivation-concentration-dispersion groups, being positioned at 2.985 Mahalanobis distance units away from quintiles 2–5 (p <0.0001) **(Table 8** and **Fig 4)**.

**Table 8:** Classification of function coefficients during a canonical discrimination analysis.

TB control indicators	Deprivation Quintiles				
	Most Deprived Quintile 1	Less Deprived Quintile 2	Less Deprived Quintile 3	Less Deprived Quintile 4	Least Deprived Quintile 5
HIV-associated TB deaths	**1.489**	1.174	1.165	.959	**.770**
(Constant)	-24.481	-16.033	-15.986	-11.330	-10.105

Fisher’s linear discriminant functions.

Standardized canonical discriminant function coefficients were used for predicting functions defining group centroids (**Fig 4**). The canonical group means (also called group centroids) are the mean for each group’s canonical observation scores. The larger the difference between the canonical groups implies the better the predictive power of the canonical discriminant function in classifying observations.

In conclusion, CDA showed that TB incidences in the most socio-economically deprived communities (quintile 1) were significantly more likely to have high HIV–associated TB death rates as compared to the least socio-economically deprived group (quintile 5) [Eigen value (12.95), function coefficient (1.49) > (.77); Wilk’s Lambda = .019, p < .0001].

## Discussion

There are two main findings of this study. First, data have shown substantial decrease of TB incidence from 2009 to 2013 due to increased healthcare expenditures, particularly expenditure per patient day equivalent (PDE) and PHC expenditure in OR Tambo district. Whilst PHC expenditure measures the total amount of money spent annually by each district divided by the total population in the district, expenditure PDE is a composite process indicator that connects financial data with service-related data from the healthcare facilities. This indicator measures how resources available to the healthcare facility are being spent, making expenditure PDE the most relevant marker of healthcare efficiency. Therefore, our findings suggest that coordinated efforts in TB control and prevention in the OR Tambo district yielded a remarkable number of new TB cases being averted.

Previous studies have shown that when healthcare expenditures are efficiently managed, TB cases will significantly decrease because health care providers will be trained to improve the ability to diagnose and treat persons with TB disease; there will be improvements in laboratory diagnostic methods with early testing and management of TB patients; implementation of appropriate infection prevention and control precautions in health care facilities and other congregate settings in order to reduce TB transmission rates; as well as strengthening of local health facilities and TB control programs in order to undertake monitoring and evaluation of TB patients on therapy [15–17].

Second, whilst milestones in reduction in TB cases are significantly progressing towards their achievements, findings from this study have unfortunately revealed that HIV associated TB deaths particularly among the most socio-economically deprived communities in OR Tambo district remain high, hence a cause for concern. The present study demonstrated that highest level of deprivation, concentration, and dispersion index was associated with the highest numbers of death among TB patients who are co-infected with HIV. Similar findings have been previously reported elsewhere [17–20].

In explaining this disparity, it is possible that either health resources are not evenly distributed between the most and the least socio-economically deprived communities to combat both TB and HIV diseases or TB and HIV health services are not fully integrated in settings where individuals are the most deprived. Either way, it is clear that TB disproportionately affects the socially and economically marginalized, with a recognized role for HIV co-infection in addition to traditional factors such as poverty and overcrowding, characterized by high population density [12, 18–21]. Since the South African provincial and local government health expenditure per headcount does provide insight into equity in justice and resources distribution [7, 10, 11], there is therefore an urgent need for addressing TB and HIV health services integration in most deprived geographical areas in the OR Tambo district in order to curb HIV associated TB deaths. Previous studies have shown that strengthening of HIV and TB services integration plays a positive role in reducing both TB/HIV incidence and mortality among individuals living under conditions of highest deprivation-concentration-dispersion [7, 16, 17, 21].

South Africa has more people living with HIV and the vast majority are on ART [9]. Despite the fact that the study by Williams demonstrated that the successful roll-out of ART has been associated with a 72% reduction in the incidence of HIV among adults from 1996 to 2016 and a 74% reduction in AIDS-related mortality from 2006 to 2016 [22], integrating TB and HIV health services is thought to actually play a major role, and might substantially reduce TB/HIV mortality in South Africa by 2030 irrespective of other traditional risk factors. Unfortunately, high rates of TB and HIV co-infection, poverty, inadequate training and supervision of the health professionals, absence of staff and material resources, and lack of infection prevention and control policies might be barriers to the implementation of HIV/TB integration, particularly among the most socio-economically deprived communities [23] such as in the O.R Tambo District as demonstrated by this present study.

## Conclusions and recommendations

The study found out that the health burden due to tuberculosis is closely correlated to expenditure per patient day equivalent, a strong indicator of healthcare efficiency. On the other hand, HIV associated TB deaths particularly among the most socio-economically deprived communities in OR Tambo district remains high. This health outcome provides a more encompassing view on the devastating effect of TB and HIV co-infection among the poorest communities. These findings highlight the importance of expanding coverage of TB and HIV integrated services to ensure prompt diagnosis and initiation of treatment among the most deprived people in resource-limited settings. Public and private partnership should be strengthening in order to develop comprehensive, collaborative and holistic approaches toward TB elimination by bringing together key stakeholders among policy makers, researchers, health professionals, civil society, non-governmental organizations (NGOs) and private sector. It is evident that that combined and coordinated efforts are the only way to halt the dual epidemics of TB and HIV by integrating healthcare services. However, further studies are warranted to address barriers and opportunities for integrating TB and HIV services among the most socio-economically deprived communities of OR Tambo district.

## Abbreviations

AFB: acid-fast bacilli
ANC: African National Congress
APP: Annual performance plan
ANOVA: Analysis of variance
CDA: canonical discriminant analysis
DC: District code
ECSECC: Eastern Cape Socio-economic Consultative Council
GDP: gross domestic product
HIV: human immunodeficiency virus
HST: Health System Trust
HSRC: Human Sciences Research Council
MDR-TB: multidrug-resistant tuberculosis
NDoH: National Department of Health
PDE: patient day equivalent
PHC: primary healthcare
SA: South Africa
SAIMD: South African Index of Multiple Deprivation
SEQ: Socio-economic quintile
SPSS: Statistical Package for the Social Sciences
TB: Tuberculosis
WHO: World Health Organization
XDR-TB: extensively-drug resistant tuberculosis
